# The crosstalk between macrophages and bone marrow mesenchymal stem cells in bone healing

**DOI:** 10.1186/s13287-022-03199-y

**Published:** 2022-11-04

**Authors:** Yu-Hao Wang, Cheng-Zhi Zhao, Ren-Yi Wang, Qian-Xin Du, Ji-Yuan Liu, Jian Pan

**Affiliations:** 1 State Key Laboratory of Oral Disease, West China Hospital of Stomatology, Sichuan University, #14 Third Section, Renmin Road South, Chengdu, 610041 People’s Republic of China; 2 National Clinical Research Center for Oral Diseases and Department of Oral and Maxillofacial Surgery, West China Hospital of Stomatology, Sichuan University, Chengdu, 610041 People’s Republic of China; 3 Chengdu Advanced Medical Science Center, West China Hospital of Stomatology, Sichuan University, Chengdu, 610041 Sichuan Province People’s Republic of China

**Keywords:** BMSCs, Macrophages, Inflammation, Tissue regeneration, Biomaterial

## Abstract

Bone injury plagues millions of patients worldwide every year, and it demands a heavy portion of expense from the public medical insurance system. At present, orthopedists think that autologous bone transplantation is the gold standard for treating large-scale bone defects. However, this method has significant limitations, which means that parts of patients cannot obtain a satisfactory prognosis. Therefore, a basic study on new therapeutic methods is urgently needed. The in-depth research on crosstalk between macrophages (Mϕs) and bone marrow mesenchymal stem cells (BMSCs) suggests that there is a close relationship between inflammation and regeneration. The in-depth understanding of the crosstalk between Mϕs and BMSCs is helpful to amplify the efficacy of stem cell-based treatment for bone injury. Only in the suitable inflammatory microenvironment can the damaged tissues containing stem cells obtain satisfactory healing outcomes. The excessive tissue inflammation and lack of stem cells make the transplantation of biomaterials necessary. We can expect that the crosstalk between Mϕs and BMSCs and biomaterials will become the mainstream to explore new methods for bone injury in the future. This review mainly summarizes the research on the crosstalk between Mϕs and BMSCs and also briefly describes the effects of biomaterials and aging on cell transplantation therapy.

## Introduction

Bones consist of 65% inorganic substance and 35% organic substance and also contain almost 99% calcium and 85% phosphorus in the whole body [1, 2]. Outer cortical bone provides considerable strength under the existence of hydroxyapatite, while inner cancellous bone provides nutrition and hematopoietic function for bones [1]. There are about 20 million people around the world to suffer from bone fractures which are mainly caused by external force or tissue lesions every year, and it brings heavy economic burdens to the public medical insurance system [3, 4]. Currently, large-scale bone defects need challenging surgical intervention to repair, such as autologous bone transplantation (the gold standard) [5, 6]. Maxillofacial surgeons usually take parts of bones from patients’ fibula to replace their diseased mandibles. However, it must be pointed out that autologous tissue transplantation will bring a series of complications to patients, which means not all patients can be guaranteed a good prognosis. Therefore, it is necessary to explore new methods for orthopedists to treat a large population with bone fractures.

Primary healing (direct method) and secondary healing (indirect method) are two healing modes for bone injury [7]. This kind of healing model is different from that of other tissues, due to the fact that there are no scar tissues formed in the whole process [8]. As the most ideal healing model for injured bones, there are three steps in secondary healing (hematoma formation, primary callus formation, and bone mineralization). The interactions among cells, signaling pathways, cytokines, and extracellular matrix are thought to be key factors to determine the prognosis of bone healing. Among the kinds of functional cells in bone healing, Mϕs and BMSCs are unique and critical [9, 10]. In the resting state, osteoblasts differentiated from BMSCs and osteoclasts differentiated from mononuclear-Mϕs keep the formation and absorption balance in bones [11].

**Fig. 1:**
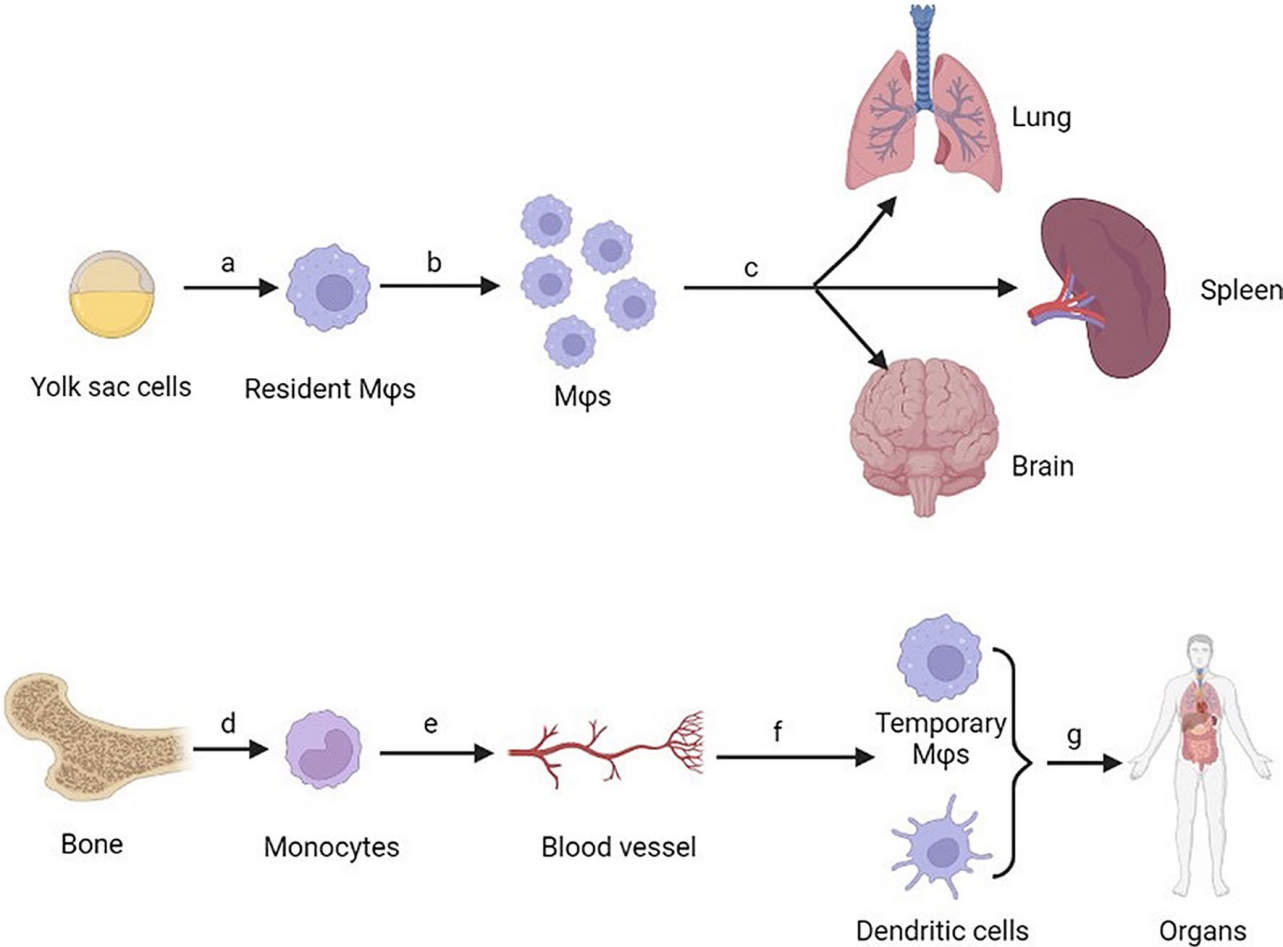
Source of Mϕs in different tissues are different. **a** Resident Mϕs are derived from yolk sac cells in embryonic stage. **b** Resident Mϕs have the ability to differentiate themselves. **c** Resident Mϕs existed in some special organs. **d** Bone tissues are able to produce large amount of monocytes. **e** Monocytes derived from bones migrate to blood vessels. **f** Monocytes have the ability to differentiate into hematopoietically derived Mϕs and dendritic cells. **g** Hematopoietically derived Mϕs and dendritic cells account for the majority of phagocytes in lots of organs in vivo

Mϕs consist of resident Mϕs derived from the yolk sac in the embryonic stage and hematopoietically derived Mϕs derived from bone marrow (Fig. 1) [12]. Resident Mϕs mainly existed in alveoli (alveolar macrophages), brain (glial cells), abdominal cavity (peritoneal macrophages), etc., and they have self-renewal capacity to keep cell population stable for life [13–15]. Hematopoietically derived Mϕs can be harvested from circulating monocytes, and mediate immune responses in target areas after being transported through blood [16]. Mϕs have the ability to eliminate cellular debris to keep the microenvironment stable by activating suitable inflammatory cascade reaction [17–20]. Recent research has shown that Mϕs would polarize toward pro-inflammatory phenotype and anti-inflammatory phenotype in the specific microenvironment [21, 22]. Wan et al. reviewed that the selective depletion of Mϕs induced in *vivo* caused long-term wound nonunion [23]. It was suggested that Mϕs not only act as inflammatory cells to eliminate necrosis components, but also as functional cells to promote tissue regeneration.

Like adipose stem cells (ASCs) in adipose tissues [24] and satellite cells in skeletal muscles [25], BMSC is a type of stem cell in bone tissues [26]. They were first isolated from human body 40 years ago and could self-proliferate and differentiate in *vitro* [27]. Flow cytometry tests show that BMSCs are CD73^+^/CD90^+^/CD105^+^/CD11b^−^/CD14^−^/CD34^−^/CD45^−^/CD19^−^/CD79a^−^/human leukocyte antigen-DR^−^ (HLA-DR^−^) [28, 29]. As progenitor cells for osteogenesis, BMSCs are able to counteract considerable degrees of bone non-pathological absorption to maintain bone structure integrity [11]. In recent years, BMSCs derived from bone marrow have been proved to exist around peripheral blood vessels and directional differentiation in targeted damaged tissues by blood transportation [26, 30–33]. Some scientists thought that cell niches in blood vessels could enhance the migration of BMSCs, which is beneficial to tissue regeneration [34].

Bone regeneration needs the cooperation of multiple systems. Among the systems that we knew, bone system and immune system are gradually considered to be vital in bone healing, due to the fact that they share many molecular regulatory networks, which creates an emerging discipline called osteoimmunology [35–37]. In conclusion, osteoimmunology studies the interactions between immune cells and bone cells in bone regeneration. In this review, we systematically retrospect the research on the crosstalk between BMSCs and Mϕs in bone healing in recent years and aim to provide new ideas for further research on bone regeneration based on cell transplantation.

## The function of Mϕs in bone healing and its regulatory role on BMSCs osteogenesis

Mϕs have been found to participate in lots of important life processes to maintain tissue homeostasis, including infection and regeneration [38, 39]. Since a long time ago, scientists generally believed that Mϕs were differentiated from monocytes derived from bone marrow and spread to target organs through the circulatory system [40–42], which is based on the differentiation of leukocytes in blood into Mϕs and dendritic cells in *vivo*. However, recent studies have found that parts of Mϕs were derived from embryonic yolk sac cells before the birth of a human instead of a myeloid source [43]. Unlike hematopoietically derived Mϕs, resident Mϕs differentiated from erythro-myeloid progenitors are able to survive for a long time and self-renewal in specific tissues. With the lack of resident Mϕs, it is generally believed that Mϕs involved in bone regeneration are derived from cancellous bones in damaged or adjacent bones.

### The fate and polarization of Mϕs

The immune response mediated by the immune system is the host's defense response to non-physiological stimulate factors, which is influenced by the expression level of inflammatory factors in the microenvironment [44]. As antigen-presenting cells in immune system, Mϕs not only phagocytize pathogens or invaded foreign bodies, but also secrete cytokines to promote or inhibit inflammation [21, 22]. After being stimulated by chemokines, monocytes migrate from bone marrow to target regions through the circulatory system and differentiate into Mϕs under the induction of macrophage colony-stimulating factor (M-CSF) and IL-4 [45]. In recent years, Mϕs are considered to be necessary for tissue regeneration in *vivo* [46–48], which is contrary to previous studies. The absence of monocytes/Mϕs inhibits osteoblast differentiation [21, 49], while the aggregation of monocytes/Mϕs induced by sphingosine-1-phosphate type I receptor agonist is able to significantly promote bone regeneration [50]. Before using Mϕs in bone regeneration, a comprehensive understanding of the fate and polarization of Mϕs is needed [51, 52].

The polarization of Mϕs determines their different function in molecular biology. Under stimulation by different cytokines, Mϕs in resting state (M_0_Mϕs) have the ability to polarize into classical activation phenotype (M_1_Mϕs) and alternative activation phenotype (M_2_Mϕs) [44, 53]. Moreover, some scholars further divided M_2_Mϕs into M_2a_/M_2b_/M_2c_ subtypes [54]. Although both M_1_Mϕs and M_2_Mϕs originate from Mϕs, they show significant differences in some aspects. Firstly, the cell morphology of M_1_Mϕs (pancake-like) and M_2_Mϕs (slender) is different [55]. Secondly, lipopolysaccharide (LPS) is necessary for Mϕs to polarize into M_1_Mϕs under the regulation of STAT1 and NF-*k*B signaling pathways [56], while Mϕs are more likely to polarize into M_2_Mϕs under the regulation of STAT6 signaling pathway when using IL-4 [55, 57]. The hardness of biomaterials is another potential factor to determine the polarization of Mϕs. For example, hydrogels with high hardness induce Mϕs to polarize into M_1_Mϕs, while Mϕs in soft hydrogels are more likely to polarize into M_2_Mϕs [58]. Xie et al*.* have thought that these polarization differences are related to cytoskeleton recombination mediated by actomyosin and actin [59], and some other scholars have thought integrins and attachment molecules on the cell surface are more important for Mϕs to identify biomaterials hardness [60, 61]. Finally, the cytokines secreted from M_1_Mϕs/M_2_Mϕs are not exactly the same. For example, current research results have shown that M_1_Mϕs mainly secrete tumor necrosis factor-α **(**TNF-α)/IL-1/IL-6/IL-12/IL-23/oncostatin M (OSM), accompanied by high expression of inducible nitric oxide synthase (iNOS)/CCR7/HLA-DR [57, 62, 63], while M_2_Mϕs mainly secrete IL-4/IL-10/IL-13/IL-1ra/vascular endothelial growth factor (VEGF)/insulin-like growth factor-1 (IGF-1), accompanied with high expression of CD163/CD206/Ym1/CCL1/CCL8/arginase-1 (Arg-1) [54, 64, 65].

The biological characteristics of M_0_Mϕs/M_1_Mϕs/M_2_Mϕs determine their roles in biological activities. He and his colleagues have found that M_1_Mϕs are able to significantly promote the proliferation and adipogenic differentiation of BMSCs, while M_0_Mϕs have a stronger ability to promote the osteogenesis of BMSCs [53]. Although in their study, M_2_Mϕs only had a weaker promotion to BMSCs for their osteogenic differentiation than that of M_0_Mϕs, they have proved that M_2_Mϕs were more likely to induce BMSCs to form thick cell sheets. M_1_Mϕs mainly secrete pro-inflammatory factors to regulate the inflammatory response. Studies have shown that sepsis is caused by a high proportion of M_1_Mϕs with excessive expression of inflammatory cytokines (IL-6/TNF-α/IL-1) [66–68]. In contrast, the cytokines secreted by M_2_Mϕs are mostly anti-inflammatory factors which are beneficial to eliminate tissue inflammation and promote tissue regeneration.

The process of inflammation and regeneration can be both found in injured tissues. In the majority of previous studies, M_1_Mϕs are mainly involved in the early inflammatory stage and M_2_Mϕs exist in the later repair stage, but some opposite views have been put forward in recent years [69–71]. For example, several studies have shown that moderate adjustment of the proportion among Mϕs subtypes could enhance the formation of vascular networks to provide enough nutrition for regenerated tissues [72–76]. Spiller et al*.* have pointed out that both M_1_Mϕs and M_2_Mϕs had the ability to secrete VEGF to promote angiogenesis, and M_2_Mϕs could also improve the secretion of platelet-derived growth factor-BB (PDGF-BB)/matrix metalloproteinase-9 (MMP-9) to enhance vascular mineralization [74]. Successful tissue regeneration requires all kinds of Mϕs to work together [77], and more details will be discussed in later chapters.

### The role of Mϕs in bone healing

In recent years, the mechanism of Mϕs in bone healing has been gradually explained, including secreting TNF-ɑ/IL-1β to promote inflammatory response and secreting bone morphogenetic protein-2 (BMP-2)/OSM/stromal cell-derived factor-1 (SDF-1)/prostaglandin2 (PGE-2)/transforming growth factor-β (TGF-β) to promote tissue regeneration [21]. One of the potential theories to explain this mechanism is that exosomes derived from Mϕs may have the ability to regulate bone homeostasis [78]. Mϕs-derived exosomes release various cytokines and miRNAs to enhance cell communication in the microenvironment [79]. In the study of Li et al*.*, exosomes derived from Mϕs could inhibit inflammation and accelerate wound healing in diabetic animal models [80]. However, some studies have pointed out that the high expression of miRNA155 could also be found in exosomes secreted by M_1_Mϕs and it was thought to inhibit vascular regeneration and aggravate cardiac dysfunction in rat models [81]. miRNA222 is another kind of small molecule found in exosomes derived from M_1_Mϕs and it acts on the anti-apoptotic gene *Bcl-2* to promote cell apoptosis (including BMSCs) [82].

Inflammatory factors secreted by M_1_Mϕs, such as TNF-α/IL-1/IL-6, mediate acute inflammatory responses after tissue injury [83]. Some scientists have thought that longer M_1_Mϕs infiltration is not conducive to tissue healing, due to the fact that it may strengthen tissue damage and influence the later regeneration processes [84–86]. However, it is worth mentioning that moderate inflammatory infiltration mediated by M_1_Mϕs is necessary and it is able to recruit stem cells into special regions to modify the microenvironment. In the study of Giannoudis et al*.*, after adding nonsteroidal anti-inflammatory drugs, the process of bone healing was inhibited or even terminated [87]. TNF-α derived from M_1_Mϕs is able to enhance immune cells’ direct killing effect on damaged cells. But Glass et al*.* have found that low concentration of TNF-α would accelerate the process of bone regeneration by increasing the expression of RUNX2, OSX, ALP, and BMP2, which meant that the effect of TNF-α on the bone healing process was time-dependent and dose-dependent [88, 89]. Furthermore, VEGF secreted by M_1_Mϕs has the potential to promote angiogenesis and make regenerated tissues have sufficient blood supply [74]. Interestingly, lots of previous studies have shown that M_1_Mϕs only participate in the early osteogenesis stage and they are absent in the later bone mineralization stage [90, 91].

Compared to M_1_Mϕs, M_2_Mϕs have a stronger ability to promote tissue regeneration [8]. Anti-inflammatory factors secreted by M_2_Mϕs, such as IL-4, can enhance tissue vascularization and inhibit the activation of osteoclasts [63, 92, 93]. In addition to inhibiting tissue inflammation, IL-4 can also promote the transformation of M_1_Mϕs to M_2_Mϕs by activating the NF-*k*B signaling pathway and continuously increase the number of M_2_Mϕs in the microenvironment [94]. IL-10 and TGF-β are other cytokines secreted by M_2_Mϕs to inhibit the inflammatory response. Similar to M_1_Mϕs, M_2_Mϕs are able to induce the formation of primary vascular structures through secreting MMP-9 and promote anastomosis between neovascularization under the regulation of PDGF-BB [74]. M_2_Mϕs secrete more BMP-2 than M_1_Mϕs, which is conducive to the osteogenic differentiation of BMSCs [95]. BMP-2 enhances the nuclear transfer of RUNX2 by activating Smad1 signaling pathway and upregulates the expression of ALP and OCN in BMSCs [91, 96]. Furthermore, M_2_Mϕs at the fracture sites do not seem to be completely polarized from Mϕs in bone marrow. For example, Doebel et al*.* have found that parts of M_2_Mϕs are migrated from the surrounding skeletal muscles in bone fracture models [97].

The existence of Mϕs has a close relationship with endochondral ossification and granulation tissue formation in injured bones [21, 98, 99]. Compared with healthy individuals, mice without Mϕs in bones show the characteristics of cartilage formation disorder and bone deposition obstruction. However, whether Mϕs are necessary for cartilage formation is still controversial, due to the fact that in the embryonic stage, the cartilage primordium already exists before the development of Mϕs lineage [100]. The ratio between M_1_Mϕs and M_2_Mϕs determines the balance between the process of inflammatory response and tissue regeneration in injured tissues [72, 101–103]. For example, the higher the proportion of M_1_Mϕs in joint synovial fluid, the more severe the symptom of arthritis is [104]. Based on the previous research, the conversion time of M_1_Mϕs to M_2_Mϕs is usually 4–7 days [105]. Schlundt et al*.* have thought that if the transformation of Mϕs was completed within 4–7 days, the later endochondral osteogenesis would not be affected anymore [99].

Although most studies have believed that Mϕs are positive and necessary in bone healing, some scholars hold the opposite view. Tang et al*.* made Mϕs and MSCs into 3D spheroids in a ratio of 1:1 for co-culture, and they found that the osteogenic ability of MSCs was significantly inhibited [106]. It may attribute to the negative role of N-cadherin in Mϕs and MSCs. However, most current studies support M_1_Mϕs and M_2_Mϕs as well as their timely transformation is indispensable in bone regeneration.

### The regulatory effect of Mϕs on the osteogenic differentiation of BMSCs

**Fig. 2:**
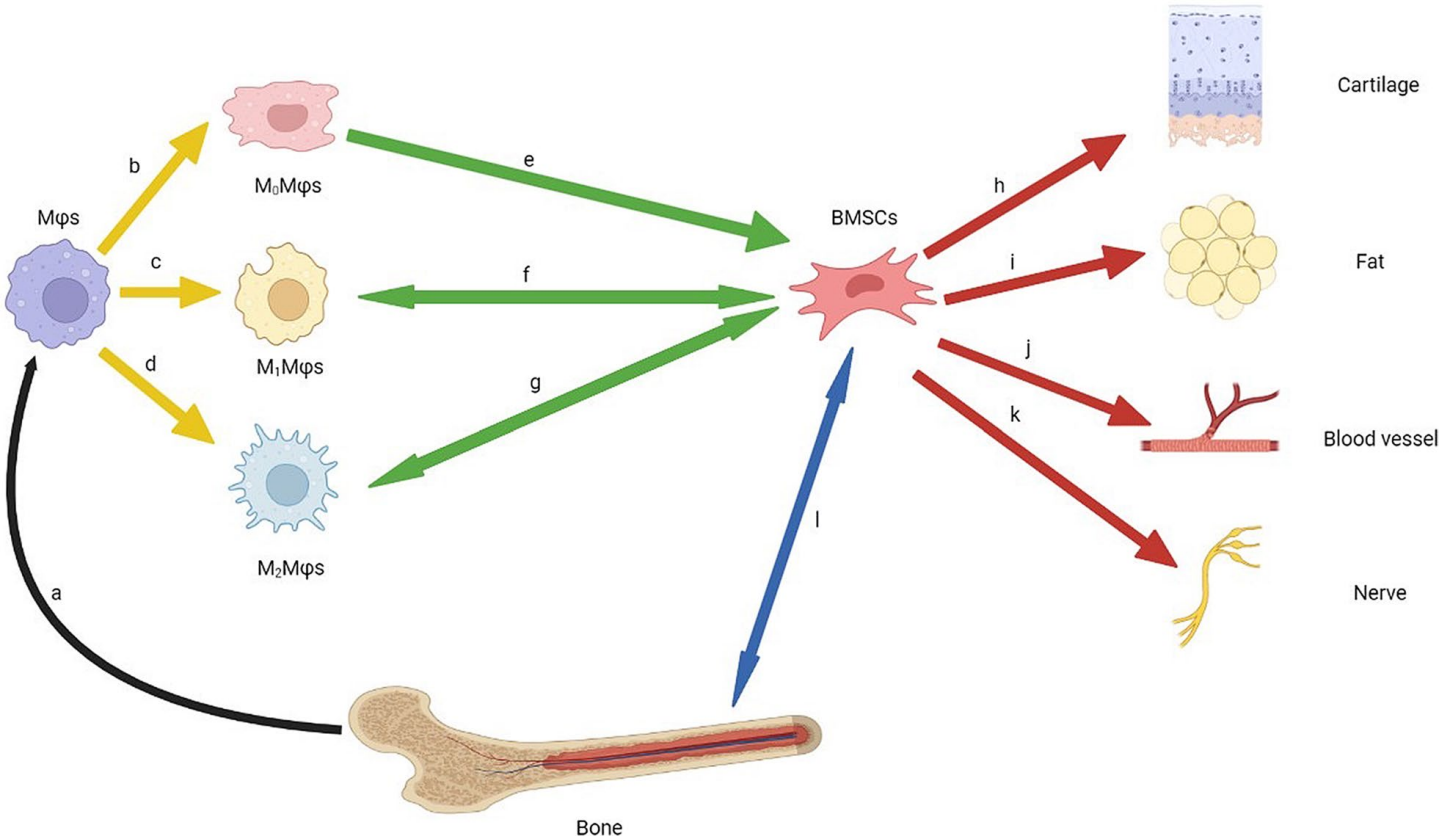
Crosstalk between Mϕs and BMSCs in bone healing. **a** Bones are able to produce Mϕs to regulate the inflammatory response. **b**–**d** Mϕs are classified into three subtypes. **e** M_0_Mϕs have a strong ability to promote the osteogenesis of BMSCs. **f** M_1_Mϕs participate in the inflammation response under the regulation of BMSCs in the early stage of bone injury. **g** M_2_Mϕs are preferred to induce BMSCs to form thick cell sheets and BMSCs are able to enhance the osteogenic promotion of M_2_Mϕs. **h**–**k** BMSCs have the ability to differentiate into various tissue cells. **l** BMSCs are the main functional cells in the process of bone healing

Mϕs not only promote bone healing, but also regulate the physiological functions of BMSCs through their paracrine effect. The extracellular matrix derived from Mϕs maintains the integrity of microenvironment and protects cell–cell or cell–matrix communication in cell sheets [107, 108]. Mϕs is important to the osteogenic differentiation of BMSCs and the crosstalk between these two kinds of cells is worth studying in the field of bone regeneration (Fig. 2).

Increasing shreds of evidence have suggested that Mϕs affect bone healing by enhancing osteogenic differentiation and migration of BMSCs [21, 109, 110]. Jin et al. have prepared an intrafibrous mineralized collagen (IMC) loaded with Mϕs-derived exosomes and implant it into rat models. They found that this kind of biomaterial was able to significantly induce the osteogenic differentiation of endogenous MSCs through Smad1/5/9 signaling pathways [111]. Extracellular matrix proteins participate in cell–cell interaction through binding integrin adhesion receptors on the cell surface, which affect the migration, differentiation, and proliferation of stem cells [112]. In addition to Smad1/5/9 signaling pathways, several studies have proved that the STAT3 signaling pathway was also positive to the regulatory effect of Mϕs on BMSCs [113]. OSM secreted by M_1_Mϕs is considered to be necessary for the osteogenic differentiation of BMSCs, which is mediated by the OSM/NF-*k*B pathway [69, 71, 114]. Furthermore, Tu et al. have proved that IL-23 derived from M_1_Mϕs also stimulates the osteogenic differentiation of BMSCs and it had relevance with the STAT3/β-catenin pathway [115]. In another experiment, when IL-23-p19 antibody was added to a co-culture medium, the osteogenic differentiation of BMSCs was significantly weakened [70], which further supported the Tu and his colleagues’ view.

M^6^A methyltransferase 3 (METTL3) has the ability to induce Mϕs to polarize into M1 phenotype, which depends on the methylation of STAT1 mRNAs [116, 117]. M_1_Mϕs are able to secrete TNF-α/IL-6/OSM/monocyte chemotactic protein-1 (MCP-1)/macrophage inflammatory protein-1α (MIP-1α) to recruit BMSCs into target regions [118, 119]. When METTL3 is selectively silenced in animal models, the secretion of these cytokines will significantly decrease and the decreasing formation of new bones will also be observed [120]. These results showed that the crosstalk between Mϕs and BMSCs may be influenced by the methylation of some specific molecular structures in Mϕs. Furthermore, some kinds of inorganic ions also play a positive role on the crosstalk between Mϕs and BMSCs. For example, when studying the osteogenic induction effect of Mg^2+^ on Mϕs, Qiao et al*.* have found an unknown mechanism about bone regeneration, including the increasing expression of CCL5/IL-8/IL-1rα and decreasing expression of IL-1β [121]. Although CCL5 and IL-8 are thought to be pro-inflammatory factors, they are able to recruit more BMSCs into damaged tissues to accelerate bone regeneration [122, 123]. Moreover, IL-8 is a key factor to promote revascularization and to keep the balance between bone reconstruction and resorption [124–126]. It should be pointed out that M_2_Mϕs-derived exosomal miRNA5106 could induce the differentiation of BMSCs by targeting salt-induced kinases 2/3 [127]. Furthermore, miRNA378a containing in M_2_Mϕs-derived exosome also promotes osteogenic differentiation of BMSCs by activating BMP signaling pathway [128].

Some studies have demonstrated that the crosstalk between Mϕs and BMSCs has no positive effect on bone regeneration. Qi et al*.* cultured Mϕs in hypoxia/serum deprivation conditions for obtaining the supernatant to inoculate with BMSCs and they found that the apoptosis rate of BMSCs in this specific medium was higher than that in the control group [129]. Then they added GM4869 into Mϕs medium to inhibit the formation of exosomes and the new supernatant had no effect on the apoptosis of BMSCs. It is indicated that the apoptosis of BMSCs may be regulated by an unknown kind of substance in Mϕs’ exosomes, and Qi et al*.* thought it should be miRNA222. In the study of He et al*.*, both M_1_Mϕs and M_2_Mϕs conditioned medium inhibited the proliferation of BMSCs. They also found that the conditioned medium of M_2_Mϕs had a stronger inhibitory effect than that of M_1_Mϕs [53].

At present, the effect of Mϕs on the osteogenic differentiation of BMSCs is still controversial, but most related research groups hold the view that M_1_Mϕs enhance osteogenesis by recruiting more BMSCs to damaged sites in the early period, while M_2_Mϕs focus more on bone matrix mineralization in the later period [95]. It requires more studies to verify Mϕs’ effect on the osteogenic differentiation of BMSCs.

### The function of BMSCs and their regulatory role on Mϕs in bone healing

Stem cells have multi-directional differentiation potential to replace damaged cells in the cell pool and maintain tissue structural integrity. Since their discovery, stem cells have been often used in cell-based therapy for various diseases. At present, there are three main strategies for stem cells to be used in clinical treatment: (a) direct transplantation; (b) pre-differentiation before transplantation; (c) obtaining cytokines derived from stem cells [130, 131]. In order to explain the function of stem cells in various tissues and cellular crosstalk between them and microenvironment, Schofield and his colleagues created a well-established concept called stem cell niche.

### The fate and biological characteristics of BMSCs

BMSCs have great potential in tissue regeneration engineering. In a previous study, Méndez-Ferrer and his colleagues used nestin to label MSCs to locate their distribution [26]. They found that although the majority of nestin^+^ MSCs located around blood vessels in the center of bone marrow, a small part of nestin^+^ MSCs could also be found near the bone intima. With low expression of HLA class I and II antigens, it is difficult for BMSCs to be captured by the host immune system [132], which significantly improve their survival rate in disease models, especially in allogeneic transplantation cases. Some scientists think that the unlimited proliferation ability of stem cells makes it impossible to use them in the clinic due to their tumorigenicity (such as iPSCs and NSCs). However, there is no obvious evidence to show that BMSCs have this disadvantage [133, 134]. BMSCs have attracted great attention in the field of tissue engineering with their easy harvest, abundant quantity, and excellent cytological characteristics.

Recently, BMSCs have been proved to promote neural regeneration [135], skeletal muscle regeneration [136], vascular regeneration [137], and bone regeneration [138]. The differentiation of BMSCs is regulated by complex molecular mechanisms. Several classical genes are thought to be pivotal signals in the multi-differentiation of BMSCs. For example, RUNX2 regulates the osteogenic differentiation of BMSCs, PPAR*γ* regulates the adipogenic differentiation of BMSCs, and Sox9 regulates the chondrogenic differentiation of BMSCs [139]. Once stimulated by the abnormal microenvironment, BMSCs will divide symmetrically or asymmetrically and migrate into injured tissues [140], which avoids excessive consumption of stem cells and ensures enough stem cells in specific regions. Furthermore, BMSCs also secrete kinds of cytokines to modify the microenvironment and eliminate inflammation, which is beneficial to accelerate the regeneration process. It is worth mentioning that any changes in the microenvironment will determine the fate of stem cells. For example, ASCs and BMSCs show obvious differences in differentiation potential, which strongly indicates that stem cells are deeply affected by their surrounding microenvironment. Recent research has demonstrated that cell–microenvironment communications are strong guarantees for stem cells to promote regeneration [107, 108].

### The role of BMSCs in bone healing

BMSCs have been proved to be able to promote tissue regeneration in auto-transplantation cases [141–145]. Hernigou et al*.* injected bone marrow aspirate into injured bones and found obvious new bone formation [146]. It is valuable for scholars to explain how BMSCs migrate from the marrow cavity to damaged sites when a bone fracture occurs. Cells in injured bones release signal molecules to increase the expression of migration-related molecules (CD44) on the BMSCs surface. BMSCs can be fixed in appropriate areas through the connection between CXCR4 on cell surface and SDF-1 (high expression in injured tissues) derived from microenvironment [110]. MMPs with a high concentration in injured sites are able to dissolve extracellular matrix to produce sufficient space to accommodate migrated BMSCs and neovascularization [147, 148]. The chemotaxis of BMSCs would be enhanced in local hypoxia caused by a vascular rupture in the early bone injury stage, due to the fact that hypoxia could enhance the production of chemokines (SDF-1/CXCR12) [149, 150].

Bone regeneration, vascular regeneration, and neural regeneration are thought to be the main steps in bone healing. Ferrer uses BMSCs marked by fluorescence to prove their differentiation ability to reconstruct the Haversian system [26]. At present, scientists generally believe that RUNX2 is vital to the osteogenic differentiation of BMSCs and it can increase the expression of osteogenic-related proteins, including integrin bone sialoprotein (IBSP) and bone gamma-carboxyglutamate protein (BGLAP) [151]. Furthermore, BMSCs are able to differentiate into endothelial cells to reconstruct capillary networks in vitro. However, the capillary formation would rarely be observed in vitro if BMSCs are cultured alone unless specific cytokines are added, such as VEGF and epidermal growth factor (EGF) [152]. It seems that BMSCs are also involved in reinnervation in regenerative bone tissues, but some scholars do not support this view. Fu et al*.* have thought that only 8% BMSCs could be induced to form neurosphere-like structures in vitro [153], while Hermann have hold the view that this proportion could be risen to 60% by unique methods [154]. The different cytokines used by different researchers are the potential reasons for different results. However, the osteogenesis and angiogenesis of BMSCs should be the basis for BMSCs to reconstruct injured bones [155].

The paracrine of BMSCs is an effective mechanism to amplify their osteogenic-promoting effect [156–159]. The organic matrix secreted by BMSCs contains type I collagen fibers, BMP, IL-1, IL-6, and osteocalcin [160]. In the study of Gao et al*.*, cytokines secreted from BMSCs promote the proliferation of endothelial cells and osteogenic differentiation of BMSCs in animal skull injury models [161]. Moreover, BMSCs upregulate the expression of VEGF to enhance vascular network formation, which is beneficial to increase oxygen supply in injured tissues [162]. However, some cytokines derived from BMSCs have been found to play a negative role in bone regeneration. For example, cytokine-like 1 (CYTL-1) was thought to inhibit the osteogenic differentiation of BMSCs by blocking RUNX2 and upregulating BAX protein expression in a recent study [163]. Shin et al*.* have proved that after being induced to osteogenic differentiation, BMSCs secrete less CYTL-1 than that in control groups in vitro, which meant that the regulatory effect of BMSCs on their osteogenic differentiation was bidirectional.

### The regulatory effect of BMSCs on the immune response mediated by Mϕs

Some scholars hold the view that BMSCs can be classified into BMSCs I phenotype (pro-inflammatory type) and BMSCs II phenotype (anti-inflammatory type) under the regulation of Toll-like receptor [164]. They can be found in different bone remodeling stages, and their target cells include Mϕs (Fig. 2). The classification of BMSCs is based on their immunomodulatory effect in bone healing. However, the academic community has not reached a consensus on this classification.

The crosstalk between BMSCs and immune cells is mediated by the connection among specific receptors on the cell surface [165, 166], which ensures that BMSCs are able to secrete anti-inflammatory factors (TGF-β and PGE2) to maintain bone immune homeostasis [21]. Furthermore, BMSCs are able to inhibit the proliferation of lymphocytes and induce the differentiation of regulatory T cells and dendritic cells. Due to their excellent anti-inflammatory properties, BMSCs have been used to eliminate inflammatory in the spinal cord injury model [167]. A recent study has shown that after being injected into myocardial infarction models, about 1% BMSCs survived and improved 3% cardiac function [168]. In myocardial infarction models, plenty of M_1_Mϕs infiltrated around cardiomyocytes in the early stage but after transplanting BMSCs, more M_2_Mϕs could be found in diseased regions [169, 170]. Therefore, under the regulation of BMSCs, the timely transformation from M_1_Mϕs to M_2_Mϕs is positive for bone healing. Cho et al*.* have found that Mϕs adjacent to BMSCs showed an increasing expression of Arg-1 which is a special marker for M_2_Mϕs [169]. It further proved that Mϕs were more likely to polarize into M_2_Mϕs co-culturing with BMSCs.

The current mainstream view is that BMSCs secrete various cytokines in the microenvironment to stimulate Mϕs to polarize into M_2_Mϕs with upregulating IL-10 and downregulating TNF-α/HLA-II/IL-12 [171]. BMSCs secrete IL-4 to promote bone regeneration by enhancing vascularization and decreasing the activation of osteoclasts [63, 92, 93]. IL-4 not only is an effective inflammatory response inhibitor, but also promotes the production of M_2_Mϕs. IL-4 is sensitive to NF-*k*B signaling pathway and has the ability to further promote BMSCs to secrete more IL-4, which forms a benign cycle to stimulate the transformation of Mϕs. However, some research results have shown that excessive secretion of IL-4 could reduce the osteogenic differentiation of BMSCs [94]. IL-1ra secreted by BMSCs is a natural inhibitor of IL-1, which blocks the connection between IL-1α and IL-1β or IL-1R through competitive binding to inhibit the polarization of Mϕs and the differentiation of dendritic cells [172].

Scientists believe that substances in exosomes derived from BMSCs activate several signaling pathways to inhibit tissue inflammation. Zhao and his colleagues have found that MSCs at the subpatellar fat pad secrete exosomes to accelerate the polarization of M_2_Mϕs by increasing the expression of Arg-1 and IL-10 [173]. In the osteoarthritis animal models, BMSCs promoted the transformation of M_1_Mϕs to M_2_Mϕs, which inhibited cartilage degradation and enhanced cartilage synthesis [174]. The miRNA223 in BMSCs’ exosomes can stimulate *pknox1* gene to induce Mϕs to polarize into M_2_Mϕs and accelerate wound healing [175]. In another study, Mao et al*.* have found that if miR-92a-3p in exosomes is overexpressed, the proliferation of chondrocytes would be enhanced with the decreasing expression of Wnt5a in tissue regeneration [176].

The regulatory effect of BMSCs on Mϕs is complex, and it involves kinds of cytokines and signaling pathways. However, it seems that the function of BMSCs on Mϕs only exists in the early stage of tissue regeneration. It is valuable for scientists to further explore how to make BMSCs continuously regulate inflammation and promote timely transformation of M_1_Mϕs to M_2_Mϕs in bone healing.

## The prospects for the crosstalk between Mϕs and BMSCs in bone healing

The ideal regeneration mode should positively regulate the hosts’ immune response to protect regenerative tissues and increase the number of stem cells [52, 177, 178]. Under the regulation of microenvironment, immune cells secrete chemokines to recruit stem cells into target regions and phagocyte pathological components in the early stage of tissue injury. The crosstalk between Mϕs and BMSCs completes the transition from tissue inflammatory to regeneration under complex mechanisms in injured tissues. The regulatory effect of Mϕs on BMSCs makes more stem cells migrate into target sites and secrete cytokines to induce Mϕs to polarization toward suitable subtypes, which is positive to accelerate bone regeneration. In summary, stem cell recruitment, cytokine secretion, and signaling pathway activating are three core elements in bone healing [155].

However, in most cases, the crosstalk between Mϕs and BMSCs in vivo is not enough to achieve the desired outcome of bone repairing due to cell deficiency, aging, and excessive tissue inflammatory. At present, the authors believe that how to enhance the crosstalk between Mϕs and BMSCs by using biomaterials and regulate the inflammatory response in the aged population will be the notable research field in the future.

### The regulatory effect of biomaterials on Mϕs and BMSCs

**Fig. 3:**
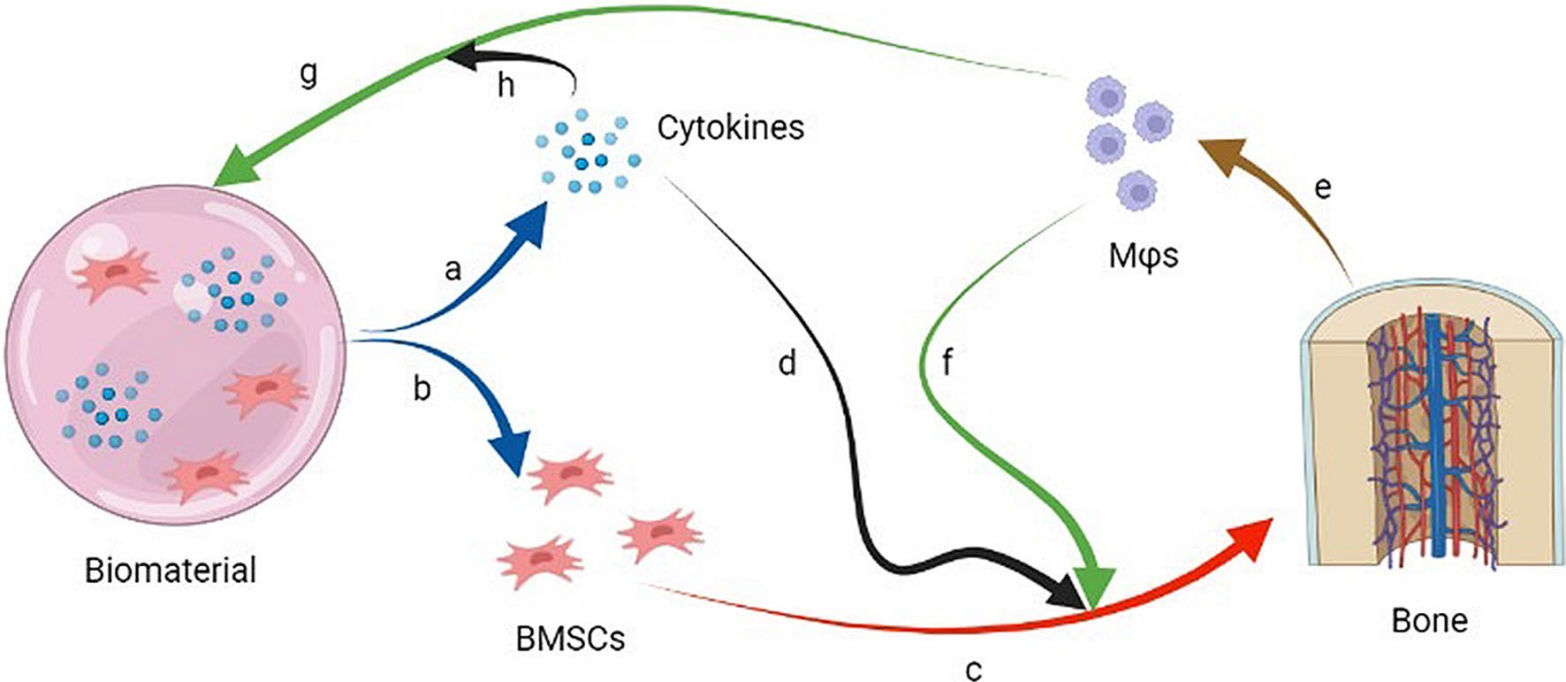
Enhancement of the crosstalk between BMSCs and Mϕs by using biomaterials will be a popular tissue engineering strategy in the future. **a**, **b** Biomaterials loaded with BMSCs and cytokines are able to release cells and cytokines into target regions. **c** BMSCs differentiate into bone cells to repair the injured bones. **d** Cytokines released from biomaterials are able to enhance the osteogenic differentiation of BMSCs. **e** Mϕs are derived from bones. **f** Mϕs participate in the osteogenic differentiation of BMSCs. **g** Biomaterials are able to protect BMSCs from the excessive inflammatory response mediated by Mϕs derived from bones. **h** Cytokines loaded on biomaterial have the ability to regulate the excessive inflammatory response

The effect of biomaterials on osteogenesis mainly depends on their biocompatibility. Biomaterials were thought to be recognized by immune system and subjected to immune attack in vivo due to their heterogeneity in the past. Foreign body reaction (FBR) and fiber wrapping often lead to unsuccessful biomaterial implantation [179, 180]. Therefore, biomaterial implantation has exactly potential negative feedback on inflammation elimination and tissue regeneration. However, the modification of biomaterials has solved the above problems and expanded their application in stem cell-based therapy. Biomaterials can be used as cell carriers to provide shelters for transplanted cells to avoid excessive infiltration, which ensures the survival of exogenous cells [181]. Furthermore, some biomaterials can slowly release anti-inflammatory factors or growth factors loaded on them in vivo and it regulates inflammation in the microenvironment to facilitate tissue regeneration. For example, in order to maintain a suitable concentration of Mg^2+^ locally, Qiao et al*.* have synthesized a kind of hydrogel which would continuously release Mg^2+^ for 7 days after implanting [121]. Perfect biomaterials must be able to stimulate moderate inflammatory response, release cytokines to nourish stem cells, and integrate new tissues (Fig. 3) [182].

The biocompatibility of biomaterials can be determined by their effects on the balance between M_1_Mϕs and M_2_Mϕs in injured tissues. Chen and his colleagues added tricalcium phosphate (TCP) particles into Mϕs medium and they observed the increasing number of M_2_Mϕs and high expression of BMP-2 in medium [183, 184]. In another study, scientists transplanted TCP particles loaded with histone methyltransferase enhancer of zeste1 (EZH1) into animal models and they found that the proportion of M_2_Mϕs was significantly increased compared with that of in the control group [44]. Moreover, another study recently has shown that β-TCP particles had the ability to enhance the osteogenic differentiation of BMSCs by inducing the polarization of Mϕs and regulating the Wnt signaling pathway [185]. However, the function of TCP particles on bone immune system is still in dispute. Some researchers added cobalt (Co) into TCP particles and found that Co-TCP composites improved the proportion of M_1_Mϕs and enhanced bone absorption [186]. The reason for different results in studies may be that the cytokines loaded with TCP particles were not the same. However, as natural components in bones, TCP particles are necessary for the physiological function of bones. In the study of Liu et al*.*, IMC had the ability to promote Mϕs to secrete functional exosomes to accelerate bone regeneration under the regulation of the BMP2/Smad5 signaling pathway [111]. Interestingly, when Mϕs co-culture with graphene oxide (Go), the inflammatory response will be strengthened and the osteogenic differentiation of BMSCs will also be enhanced by activating OSM/NF-*k*B signaling pathway [69, 71]. Ujiie has designed a kind of scaffold materials with releasing interferon-γ (IFN-γ)/IL-4 in sequence. They also proved that it was positive for the polarization of Mϕs and revascularization in regenerative tissues [187].

The regulatory effect of biomaterials on BMSCs also profoundly affects the fate of Mϕs. Gamblin et al. implanted BMSCs on biphasic calcium phosphate (BCP) particles to construct a new BMSCs-biomaterial system and observed obvious chemotaxis of Mϕs [188, 189]. Xue et al. have found that after inoculating BMSCs in Cu-MSN/Mϕs conditioned medium, the osteoprotegerin (OPG) secreted by BMSCs was significantly upregulated accompanied by the downregulation of RANKL, both of which were positive for bone regeneration [69]. In other several studies, scholars transplanted fibrin and hydroxyapatite as cell carriers into experimental animals and found that the majority of Mϕs polarized into M_1_Mϕs in the early stage, but it is interesting that M_1_Mϕs would be transformed into M_2_Mϕs in the later stage. The reason for it may be that exogenous BMSCs secreted enough PEG to activate the NF-*k*B signaling pathway under the regulation of microenvironment [189–191].

The physicochemical properties of biomaterials also have a far-reaching impact on the crosstalk between BMSCs and Mϕs [192]. As mentioned above, the hardness of biomaterial influence the polarization of Mϕs. The hard materials induce the production of M_1_Mϕs, while soft materials induce the production of M_2_Mϕs. Scientists cultured Mϕs derived from bone marrow on hard polyethylene glycol hydrogel and they found that Mϕs expressed more TNF-α and IL-1β than that of in soft material group [193]. However, the hardness of biomaterials has the opposite effects on the osteogenic differentiation of BMSCs. Several studies have shown that without other interference factors, hard biomaterials were beneficial to the osteogenic differentiation of BMSCs [194, 195]. Therefore, it is necessary to deeply study the effect of biomaterial hardness on the crosstalk between BMSCs and Mϕs. The porous structures in biomaterials are channels to transport nutritional factors and oxygen for loaded cells. In a previous study, a kind of porous biomaterial (the diameter of the micropore was 34 µm) could significantly increase the number of M_1_Mϕs in rat models and have a negative effect on bone healing [196]. Electrical stimulation is able to change the migration, phagocytosis, and cytokine production of Mϕs, which provides unexpected benefits for bone healing [197, 198]. Under the stimulation of microcurrent, M_1_Mϕs cultured in high glucose medium would downregulate the transduction of AKT2-IRF5 signal and terminate the process of polarization [199]. Furthermore, the maturity of micro–nanotechnology makes it possible to modify biomaterial surface to improve cell activities, including adding groove structures [176].

### The aging effects on Mϕs and BMSCs

The typical characteristic of aging is the functional degradation of tissues and cells. Aging has obvious effects on all kinds of cells, especially adult stem cells and Mϕs. Mahbub et al*.* have found that aged M_1_Mϕs secreted less IL-1β and TNF-α than younger ones, which caused a mild inflammatory reaction when tissues were injured [200]. In contrast, another study conducted by Brrett has demonstrated that aged M_1_Mϕs mediated more severe inflammatory response and prolonged inflammation process [201]. The latter view seems to be supported by some research that focused on this topic. For example, Gibon and his colleagues have proved that aged M_1_Mϕs significantly upregulated the expression of pro-inflammatory factor (TNF-α) and down-regulated the expression of anti-inflammatory factor (IL-1ra). It also explained why tissues in the elderly were always in a high inflammatory state [201, 202]. Furthermore, after interconnecting the circulatory system of aged rats with young rats, inflammatory response inhibition and bone regeneration acceleration could be clearly seen in aged groups [203].

BMSCs were more sensitive to aging than Mϕs. With age increasing, the weak biological function and apoptosis of stem cells slow down the tissue regeneration process. Fat tissues will gradually accumulate in the bone marrow cavity to affect the osteogenic differentiation ability of BMSCs in aged groups [204]. In contrast, the adipogenic capacity of BMSCs will gradually enhance with aging, which also weakens the process of bone regeneration [205]. The increasing adipogenic capacity of BMSCs with aging may be attributed to the decreased expression of transcriptional coactivators containing PDZ binding sequences, which leads to the increasing expression of PPAR*γ* and decreasing expression of RUNX2 [206]. The low metabolic capacity and strong adipogenic capacity of BMSCs in elderly individuals lead to a large accumulation of adipose tissues in bone marrow, which brings a fatal blow to the self-healing in patients with fracture.

Aging makes old patients not only suffer from more pain during fracture healing, but also get the worse healing outcome than young patients. How to solve this problem is another aim for scientists in the field of tissue engineering.

## Conclusion

In recent years, studies on crosstalk between Mϕs and BMSCs have suggested that the bone healing process is complex. Inflammatory mediated by Mϕs plays a vital role in bone regeneration. On the one hand, excessive inflammatory responses may cause irreversible tissue damage and affect adjacent healthy tissues. On the other hand, mild inflammatory responses activate signaling pathways that are related to tissue regeneration to repair damaged tissues. The inflammatory response degree depends on the proportion and transformation of M_1_Mϕs and M_2_Mϕs in the microenvironment. BMSCs are another important kind of cells in bone healing. Both the osteogenic differentiation of BMSCs and cytokines derived from BMSCs are necessary to bone regeneration. The effect of crosstalk between Mϕs and BMSCs is significant to the outcome of bone healing. The inflammatory mediated by Mϕs is able to regulate the osteogenic differentiation of BMSCs, and meanwhile, the cytokines secreted by BMSCs are also able to inhibit or stimulate the inflammatory response in bones. Therefore, more studies are needed to focus on how to regulate the crosstalk between BMSCs and Mϕs to accelerate bone healing.

The recent research on biomaterials provides us with new methods to solve problems about the insufficient number of stem cells and excessive inflammatory responses. The stem cell–biomaterial models are not only able to carry seed cells, but also to load with various necessary cytokines, which significantly modify the microenvironment in damaged tissues. The emergence of biomaterials can also effectively improve prognosis due to high inflammatory status of tissues in elder patients. The development of new biomaterials and modification of existing biomaterials are effective methods to improve tissue regeneration, and it demands more research for further exploration and discussion.

## Data Availability

Not applicable.

## Abbreviations

Arg-1: Arginase-1
ASCs: Adipose stem cells
BCP: Biphasic calcium phosphate
BGLAP: Bone gamma-carboxyglutamate protein
BMP-2: Bone morphogenetic protein-2
BMSCs: Bone marrow mesenchymal stem cells
Co: Cobalt
CYTL-1: Cytokine-like 1
EGF: Epidermal growth factor
EZH1: Histone methyltransferase enhancer of zestel 1
FBR: Foreign body reaction
Go: Graphene oxide
HLA-DR: Human leukocyte antigen-DR
IBSP: Integrin bone sialoprotein
IFN-γ: Interferon-γ
IGF-1: Insulin-like growth factor-1
IMC: Intrafibrous mineralized collagen
LPS: Lipopolysaccharide
Mϕs: Macrophages
MCP-1: Monocyte chemotactic protein-1
M-CSF: Macrophage colony-stimulating factor
METTL3: M^6^A methyltransferase 3
MMP-9: Matrix metalloproteinase-9
MIP-1α: Macrophage inflammatory protein-1α
OPG: Osteoprotegerin
OSM: Oncostatin M
PDGF-BB: Platelet-derived growth factor-BB
PGE2: Prostaglandin2
SDF-1: Stromal cell-derived factor-1
TCP: Tricalcium phosphate
TGF-β: Transforming growth factor-β
TNF-α: Tumor necrosis factor-α
VEGF: Vascular endothelial growth factor

## References

[1] Buck DW, Dumanian GA (2012). Bone biology and physiology: Part I. The fundamentals. Plast Reconstr Surg.

[2] Bae WC, Chen PC, Chung CB, Masuda K, D'Lima D, Du J (2012). Quantitative ultrashort echo time (UTE) MRI of human cortical bone: correlation with porosity and biomechanical properties. J Bone Miner Res.

[3] Habibovic P (2017). * Strategic directions in osteoinduction and biomimetics. Tissue Eng Part A.

[4] Iaquinta MR, Mazzoni E, Bononi I, Rotondo JC, Mazziotta C, Montesi M, Sprio S, Tampieri A, Tognon M, Martini F (2019). Adult stem cells for bone regeneration and repair. Front Cell Dev Biol.

[5] Pessoa EAM, Braune A, Casado PL, Tannure PN (2017). Alveolar bone graft: clinical profile and risk factors for complications in oral cleft patients. Cleft Palate Craniofac J.

[6] De Long WG, Einhorn TA, Koval K, McKee M, Smith W, Sanders R, Watson T (2007). Bone grafts and bone graft substitutes in orthopaedic trauma surgery: a critical analysis. J Bone Joint Surg Am.

[7] Dimitriou R, Jones E, McGonagle D, Giannoudis PV (2011). Bone regeneration: current concepts and future directions. BMC Med.

[8] Shin RL, Lee CW, Shen OY, Xu H, Lee OK (2021). The crosstalk between mesenchymal stem cells and macrophages in bone regeneration: a systematic review. Stem Cells Int.

[9] Zhu Y, Deng S, Ma Z, Kong L, Li H, Chan HF (2021). Macrophages activated by akermanite/alginate composite hydrogel stimulate migration of bone marrow-derived mesenchymal stem cells. Biomed Mater.

[10] Baht GS, Vi L, Alman BA (2018). The role of the immune cells in fracture healing. Curr Osteoporos Rep.

[11] Font Tellado S, Balmayor ER, Van Griensven M (2015). Strategies to engineer tendon/ligament-to-bone interface: Biomaterials, cells and growth factors. Adv Drug Deliv Rev.

[12] Perdiguero EG, Geissmann F (2016). The development and maintenance of resident macrophages. Nat Immunol.

[13] Davies LC, Rosas M, Smith PJ, Fraser DJ, Jones SA, Taylor PR (2011). A quantifiable proliferative burst of tissue macrophages restores homeostatic macrophage populations after acute inflammation. Eur J Immunol.

[14] Ghigo C, Mondor I, Jorquera A, Nowak J, Wienert S, Zahner SP, Clausen BE, Luche H, Malissen B, Klauschen F, Bajénoff M (2013). Multicolor fate mapping of Langerhans cell homeostasis. J Exp Med.

[15] Kanitakis J, Morelon E, Petruzzo P, Badet L, Dubernard JM (2011). Self-renewal capacity of human epidermal Langerhans cells: observations made on a composite tissue allograft. Exp Dermatol.

[16] van Furth R, Cohn ZA (1968). The origin and kinetics of mononuclear phagocytes. J Exp Med.

[17] Michalski MN, Koh AJ, Weidner S, Roca H, McCauley LK (2016). Modulation of Osteoblastic cell efferocytosis by bone marrow macrophages. J Cell Biochem.

[18] Huynh ML, Fadok VA, Henson PM (2002). Phosphatidylserine-dependent ingestion of apoptotic cells promotes TGF-beta1 secretion and the resolution of inflammation. J Clin Invest.

[19] Xiao YQ, Freire-de-Lima CG, Schiemann WP, Bratton DL, Vandivier RW, Henson PM (2008). Transcriptional and translational regulation of TGF-beta production in response to apoptotic cells. J Immunol.

[20] Song X, Xue Y, Fan S, Hao J, Deng R (2022). Lipopolysaccharide-activated macrophages regulate the osteogenic differentiation of bone marrow mesenchymal stem cells through exosomes. PeerJ.

[21] Vi L, Baht GS, Whetstone H, Ng A, Wei Q, Poon R, Mylvaganam S, Grynpas M, Alman BA (2015). Macrophages promote osteoblastic differentiation in-vivo: implications in fracture repair and bone homeostasis. J Bone Miner Res.

[22] Miron RJ, Bosshardt DD (2016). OsteoMacs: key players around bone biomaterials. Biomaterials.

[23] Wan Z, Shin LY, Wang YF, Huang Z, Dong Y, Lee CW, Kumta SM, Lee OK (2020). Role of skeletal macrophages in fracture repair: a systematic review. Biomed Rep.

[24] Wang YH, Guo YC, Wang DR, Liu JY, Pan J (2019). Adipose stem cell-based clinical strategy for neural regeneration: a review of current opinion. Stem Cells Int.

[25] Wang YH, Wang DR, Guo YC, Liu JY, Pan J (2020). The application of bone marrow mesenchymal stem cells and biomaterials in skeletal muscle regeneration. Regen Ther.

[26] Méndez-Ferrer S, Michurina TV, Ferraro F, Mazloom AR, Macarthur BD, Lira SA, Scadden DT, Ma'ayan A, Enikolopov GN, Frenette PS (2010). Mesenchymal and haematopoietic stem cells form a unique bone marrow niche. Nature.

[27] Friedenstein AJ, Chailakhjan RK, Lalykina KS (1970). The development of fibroblast colonies in monolayer cultures of guinea-pig bone marrow and spleen cells. Cell Tissue Kinet.

[28] Dominici M, Le Blanc K, Mueller I, Slaper-Cortenbach I, Marini F, Krause D, Deans R, Keating A, Prockop D, Horwitz E (2006). Minimal criteria for defining multipotent mesenchymal stromal cells: The International Society for Cellular Therapy position statement. Cytotherapy.

[29] Boxall SA, Jones E (2012). Markers for characterization of bone marrow multipotential stromal cells. Stem Cells Int.

[30] Wang Y, Pan J, Wang D, Liu J (2018). The use of stem cells in neural regeneration: a review of current opinion. Curr Stem Cell Res Ther.

[31] Caplan AI, Correa D (2011). The MSC: an injury drugstore. Cell Stem Cell.

[32] Hoshino A, Chiba H, Nagai K, Ishii G, Ochiai A (2008). Human vascular adventitial fibroblasts contain mesenchymal stem/progenitor cells. Biochem Biophys Res Commun.

[33] de Souza LE, Malta TM, Kashima Haddad S, Covas DT (2016). Mesenchymal stem cells and pericytes: To what extent are they related?. Stem Cells Dev.

[34] Kuhn NZ, Tuan RS (2010). Regulation of stemness and stem cell niche of mesenchymal stem cells: implications in tumorigenesis and metastasis. J Cell Physiol.

[35] Okamoto K, Nakashima T, Shinohara M, Negishi-Koga T, Komatsu N, Terashima A, Sawa S, Nitta T, Takayanagi H (2017). Osteoimmunology: The conceptual framework unifying the immune and skeletal systems. Physiol Rev.

[36] Takayanagi H (2007). Osteoimmunology: shared mechanisms and crosstalk between the immune and bone systems. Nat Rev Immunol.

[37] Dar HY, Azam Z, Anupam R, Mondal RK, Srivastava RK (2018). Osteoimmunology: the *Nexus* between bone and immune system. Front Biosci (Landmark Ed).

[38] Murray PJ, Wynn TA (2011). Protective and pathogenic functions of macrophage subsets. Nat Rev Immunol.

[39] Wynn TA, Barron L (2010). Macrophages: master regulators of inflammation and fibrosis. Semin Liver Dis.

[40] Liu K, Victora GD, Schwickert TA, Guermonprez P, Meredith MM, Yao K, Chu FF, Randolph GJ, Rudensky AY, Nussenzweig M (2009). In vivo analysis of dendritic cell development and homeostasis. Science.

[41] Auffray C, Fogg DK, Narni-Mancinelli E, Senechal B, Trouillet C, Saederup N, Leemput J, Bigot K, Campisi L, Abitbol M, Molina T, Charo I, Hume DA, Cumano A, Lauvau G, Geissmann F (2009). CX3CR1+ CD115+ CD135+ common macrophage/DC precursors and the role of CX3CR1 in their response to inflammation. J Exp Med.

[42] Liu K, Waskow C, Liu X, Yao K, Hoh J, Nussenzweig M (2007). Origin of dendritic cells in peripheral lymphoid organs of mice. Nat Immunol.

[43] Geissmann F, Manz MG, Jung S, Sieweke MH, Merad M, Ley K (2010). Development of monocytes, macrophages, and dendritic cells. Science.

[44] Jia X, Xu H, Miron RJ, Yin C, Zhang X, Wu M, Zhang Y (2018). EZH1 is associated with TCP-induced bone regeneration through macrophage polarization. Stem Cells Int.

[45] Gong F, Groth T, Tu C, Zhao M, Chu J (2021). Crosstalk between macrophages and mesenchymal stem cells regulated by biomaterials and its role in bone regeneration. Adv Mater Sci Eng.

[46] Katagiri W, Takeuchi R, Saito N, Suda D, Kobayashi T (2022). Migration and phenotype switching of macrophages at early-phase of bone-formation by secretomes from bone marrow derived mesenchymal stem cells using rat calvaria bone defect model. J Dent Sci.

[47] Zhang W, Zhao F, Huang D, Fu X, Li X, Chen X (2016). Strontium-substituted submicrometer bioactive glasses modulate macrophage responses for improved bone regeneration. ACS Appl Mater Interfaces.

[48] Sadtler K, Estrellas K, Allen BW, Wolf MT, Fan H, Tam AJ, Patel CH, Luber BS, Wang H, Wagner KR, Powell JD, Housseau F, Pardoll DM, Elisseeff JH (2016). Developing a pro-regenerative biomaterial scaffold microenvironment requires T helper 2 cells. Science.

[49] Jin SS, He DQ, Luo D, Wang Y, Yu M, Guan B, Fu Y, Li ZX, Zhang T, Zhou YH, Wang CY, Liu Y (2019). A biomimetic hierarchical nanointerface orchestrates macrophage polarization and mesenchymal stem cell recruitment to promote endogenous bone regeneration. ACS Nano.

[50] Kim YH, Furuya H, Tabata Y (2014). Enhancement of bone regeneration by dual release of a macrophage recruitment agent and platelet-rich plasma from gelatin hydrogels. Biomaterials.

[51] Franz S, Rammelt S, Scharnweber D, Simon JC (2011). Immune responses to implants: a review of the implications for the design of immunomodulatory biomaterials. Biomaterials.

[52] Yu Y, Wu RX, Yin Y, Chen FM (2016). Directing immunomodulation using biomaterials for endogenous regeneration. J Mater Chem B.

[53] He XT, Li X, Yin Y, Wu RX, Xu XY, Chen FM (2018). The effects of conditioned media generated by polarized macrophages on the cellular behaviours of bone marrow mesenchymal stem cells. J Cell Mol Med.

[54] Lawrence T, Natoli G (2011). Transcriptional regulation of macrophage polarization: enabling diversity with identity. Nat Rev Immunol.

[55] Pajarinen J, Lin TH, Sato T, Loi F, Yao Z, Konttinen YT, Goodman SB (2015). Establishment of green fluorescent protein and firefly luciferase expressing mouse primary macrophages for in vivo bioluminescence imaging. PLoS ONE.

[56] Lin TH, Tamaki Y, Pajarinen J, Waters HA, Woo DK, Yao Z, Goodman SB (2014). Chronic inflammation in biomaterial-induced periprosthetic osteolysis: NF-κB as a therapeutic target. Acta Biomater.

[57] Murray PJ, Allen JE, Biswas SK, Fisher EA, Gilroy DW, Goerdt S, Gordon S, Hamilton JA, Ivashkiv LB, Lawrence T, Locati M, Mantovani A, Martinez FO, Mege JL, Mosser DM, Natoli G, Saeij JP, Schultze JL, Shirey KA, Sica A, Suttles J, Udalova I, van Ginderachter JA, Vogel SN, Wynn TA (2014). Macrophage activation and polarization: nomenclature and experimental guidelines. Immunity.

[58] Sridharan R, Cavanagh B, Cameron AR, Kelly DJ, O'Brien FJ (2019). Material stiffness influences the polarization state, function and migration mode of macrophages. Acta Biomater.

[59] Xie J, Bao M, Hu X, Koopman WJH, Huck WTS (2021). Energy expenditure during cell spreading influences the cellular response to matrix stiffness. Biomaterials.

[60] Leckband DE, le Duc Q, Wang N, de Rooij J (2011). Mechanotransduction at cadherin-mediated adhesions. Curr Opin Cell Biol.

[61] Geiger B, Bershadsky A, Pankov R, Yamada KM (2001). Transmembrane crosstalk between the extracellular matrix–cytoskeleton crosstalk. Nat Rev Mol Cell Biol.

[62] Guihard P, Boutet MA, Brounais-Le Royer B, Gamblin AL, Amiaud J, Renaud A, Berreur M, Rédini F, Heymann D, Layrolle P, Blanchard F (2015). Oncostatin m, an inflammatory cytokine produced by macrophages, supports intramembranous bone healing in a mouse model of tibia injury. Am J Pathol.

[63] Spiller KL, Nassiri S, Witherel CE, Anfang RR, Ng J, Nakazawa KR, Yu T, Vunjak-Novakovic G (2015). Sequential delivery of immunomodulatory cytokines to facilitate the M1-to-M2 transition of macrophages and enhance vascularization of bone scaffolds. Biomaterials.

[64] Nair MG, Du Y, Perrigoue JG, Zaph C, Taylor JJ, Goldschmidt M, Swain GP, Yancopoulos GD, Valenzuela DM, Murphy A, Karow M, Stevens S, Pearce EJ, Artis D (2009). Alternatively activated macrophage-derived RELM-{alpha} is a negative regulator of type 2 inflammation in the lung. J Exp Med.

[65] Maresz K, Ponomarev ED, Barteneva N, Tan Y, Mann MK, Dittel BN (2008). IL-13 induces the expression of the alternative activation marker Ym1 in a subset of testicular macrophages. J Reprod Immunol.

[66] Prockop DJ, Oh JY (2012). Mesenchymal stem/stromal cells (MSCs): role as guardians of inflammation. Mol Ther.

[67] Lai X, Ding H, Yu R, Bai H, Liu Y, Cao J (2022). CXCL14 protects against polymicrobial sepsis by enhancing antibacterial functions of macrophages. Am J Respir Cell Mol Biol.

[68] Liu LL, Gong LK, Wang H, Xiao Y, Wu XF, Zhang YH, Xue X, Qi XM, Ren J (2008). Baicalin inhibits macrophage activation by lipopolysaccharide and protects mice from endotoxin shock. Biochem Pharmacol.

[69] Xue D, Chen E, Zhong H, Zhang W, Wang S, Joomun MU, Yao T, Tan Y, Lin S, Zheng Q, Pan Z (2018). Immunomodulatory properties of graphene oxide for osteogenesis and angiogenesis. Int J Nanomedicine.

[70] Wasnik S, Rundle CH, Baylink DJ, Yazdi MS, Carreon EE, Xu Y, Qin X, Lau KW, Tang X (2018). 1,25-Dihydroxyvitamin D suppresses M1 macrophages and promotes M2 differentiation at bone injury sites. JCI Insight.

[71] Hirata E, Miyako E, Hanagata N, Ushijima N, Sakaguchi N, Russier J, Yudasaka M, Iijima S, Bianco A, Yokoyama A (2016). Carbon nanohorns allow acceleration of osteoblast differentiation via macrophage activation. Nanoscale.

[72] Boersema GS, Grotenhuis N, Bayon Y, Lange JF, Bastiaansen-Jenniskens YM (2016). The effect of biomaterials used for tissue regeneration purposes on polarization of macrophages. Biores Open Access.

[73] Ma QL, Zhao LZ, Liu RR, Jin BQ, Song W, Wang Y, Zhang YS, Chen LH, Zhang YM (2014). Improved implant osseointegration of a nanostructured titanium surface via mediation of macrophage polarization. Biomaterials.

[74] Spiller KL, Anfang RR, Spiller KJ, Ng J, Nakazawa KR, Daulton JW, Vunjak-Novakovic G (2014). The role of macrophage phenotype in vascularization of tissue engineering scaffolds. Biomaterials.

[75] Wu RX, Yin Y, He XT, Li X, Chen FM (2017). Engineering a cell home for stem cell homing and accommodation. Adv Biosyst.

[76] Spiller KL, Freytes DO, Vunjak-Novakovic G (2015). Macrophages modulate engineered human tissues for enhanced vascularization and healing. Ann Biomed Eng.

[77] Nathan K, Lu LY, Lin T, Pajarinen J, Jämsen E, Huang JF, Romero-Lopez M, Maruyama M, Kohno Y, Yao Z, Goodman SB (2019). Precise immunomodulation of the M1 to M2 macrophage transition enhances mesenchymal stem cell osteogenesis and differs by sex. Bone Joint Res.

[78] Golchin A, Hosseinzadeh S, Ardeshirylajimi A (2018). The exosomes released from different cell types and their effects in wound healing. J Cell Biochem.

[79] Zhang D, Lee H, Wang X, Rai A, Groot M, Jin Y (2018). Exosome-mediated small RNA delivery: a novel therapeutic approach for inflammatory lung responses. Mol Ther.

[80] Li M, Wang T, Tian H, Wei G, Zhao L, Shi Y (2019). Macrophage-derived exosomes accelerate wound healing through their anti-inflammation effects in a diabetic rat model. Artif Cells Nanomed Biotechnol.

[81] Liu S, Chen J, Shi J, Zhou W, Wang L, Fang W, Zhong Y, Chen X, Chen Y, Sabri A, Liu S (2020). M1-like macrophage-derived exosomes suppress angiogenesis and exacerbate cardiac dysfunction in a myocardial infarction microenvironment. Basic Res Cardiol.

[82] Wang W, Wang J, Zhang J, Taq W, Zhang Z (2019). miR-222 induces apoptosis in human intervertebral disc nucleus pulposus cells by targeting Bcl-2. Mol Med Rep.

[83] Marsell R, Einhorn TA (2011). The biology of fracture healing. Injury.

[84] Lienau J, Schmidt-Bleek K, Peters A, Haschke F, Duda GN, Perka C, Bail HJ, Schütze N, Jakob F, Schell H (2009). Differential regulation of blood vessel formation between standard and delayed bone healing. J Orthop Res.

[85] Zhang R, Liu X, Xiong Z, Huang Q, Yang X, Yan H, Ma J, Feng Q, Shen Z (2018). The immunomodulatory effects of Zn-incorporated micro/nanostructured coating in inducing osteogenesis. Artif Cells Nanomed Biotechnol.

[86] Claes L, Recknagel S, Ignatius A (2012). Fracture healing under healthy and inflammatory conditions. Nat Rev Rheumatol.

[87] Giannoudis PV, MacDonald DA, Matthews SJ, Smith RM, Furlong AJ, De Boer P (2000). Nonunion of the femoral diaphysis: The influence of reaming and non-steroidal anti-inflammatory drugs. J Bone Joint Surg Br.

[88] Glass GE, Chan JK, Freidin A, Feldmann M, Horwood NJ, Nanchahal J (2011). TNF-alpha promotes fracture repair by augmenting the recruitment and differentiation of muscle-derived stromal cells. Proc Natl Acad Sci USA.

[89] Huang H, Zhao N, Xu X, Xu Y, Li S, Zhang J, Yang P (2011). Dose-specific effects of tumor necrosis factor alpha on osteogenic differentiation of mesenchymal stem cells. Cell Prolif.

[90] Vergadi E, Ieronymaki E, Lyroni K, Vaporidi K, Tsatsanis C (2017). Akt signaling pathway in macrophage activation and M1/M2 polarization. J Immunol.

[91] Pajarinen J, Lin T, Gibon E, Kohno Y, Maruyama M, Nathan K, Lu L, Yao Z, Goodman SB (2019). Mesenchymal stem cell-macrophage crosstalk and bone healing. Biomaterials.

[92] Loi F, Córdova LA, Pajarinen J, Lin TH, Yao Z, Goodman SB (2016). Inflammation, fracture and bone repair. Bone.

[93] Lin T, Pajarinen J, Nabeshima A, Lu L, Nathan K, Yao Z, Goodman SB (2017). Establishment of NF-κB sensing and interleukin-4 secreting mesenchymal stromal cells as an "on-demand" drug delivery system to modulate inflammation. Cytotherapy.

[94] Wei F, Li Z, Crawford R, Xiao Y, Zhou Y (2019). Immunoregulatory role of exosomes derived from differentiating mesenchymal stromal cells on inflammation and osteogenesis. J Tissue Eng Regen Med.

[95] Zhang Y, Böse T, Unger RE, Jansen JA, Kirkpatrick CJ, van den Beucken JJJP (2017). Macrophage type modulates osteogenic differentiation of adipose tissue MSCs. Cell Tissue Res.

[96] Muñoz J, Akhavan NS, Mullins AP, Arjmandi BH (2020). Macrophage polarization and osteoporosis: A Review. Nutrients.

[97] Doebel T, Voisin B, Nagao K (2017). Langerhans cells: the macrophage in dendritic cell clothing. Trends Immunol.

[98] Raggatt LJ, Wullschleger ME, Alexander KA, Wu AC, Millard SM, Kaur S, Maugham ML, Gregory LS, Steck R, Pettit AR (2014). Fracture healing via periosteal callus formation requires macrophages for both initiation and progression of early endochondral ossification. Am J Pathol.

[99] Schlundt C, El Khassawna T, Serra A, Dienelt A, Wendler S, Schell H, van Rooijen N, Radbruch A, Lucius R, Hartmann S, Duda GN, Schmidt-Bleek K (2018). Macrophages in bone fracture healing: their essential role in endochondral ossification. Bone.

[100] Lichanska AM, Browne CM, Henkel GW, Murphy KM, Ostrowski MC, McKercher SR, Maki RA, Hume DA (1999). Differentiation of the mononuclear phagocyte system during mouse embryogenesis: the role of transcription factor PU.1. Blood.

[101] Mantovani A, Sica A, Sozzani S, Allavena P, Vecchi A, Locati M (2004). The chemokine system in diverse forms of macrophage activation and polarization. Trends Immunol.

[102] Wynn TA, Chawla A, Pollard JW (2013). Macrophage biology in development, homeostasis and disease. Nature.

[103] Brown BN, Badylak SF (2013). Expanded applications, shifting paradigms and an improved understanding of host-biomaterial interactions. Acta Biomater.

[104] Rőszer T (2015). Understanding the mysterious M2 macrophage through activation markers and effector mechanisms. Mediators Inflamm.

[105] Spriano S, Yamaguchi S, Baino F, Ferraris S (2018). A critical review of multifunctional titanium surfaces: new frontiers for improving osseointegration and host response, avoiding bacteria contamination. Acta Biomater.

[106] Tang H, Husch JFA, Zhang Y, Jansen JA, Yang F, van den Beucken JJJP (2019). Coculture with monocytes/macrophages modulates osteogenic differentiation of adipose-derived mesenchymal stromal cells on poly(lactic-co-glycolic) acid/polycaprolactone scaffolds. J Tissue Eng Regen Med.

[107] Bonnans C, Chou J, Werb Z (2014). Remodelling the extracellular matrix in development and disease. Nat Rev Mol Cell Biol.

[108] Chen FM, Liu X (2016). Advancing biomaterials of human origin for tissue engineering. Prog Polym Sci.

[109] Lo CH, Lynch CC (2018). Multifaceted roles for macrophages in prostate cancer skeletal metastasis. Front Endocrinol (Lausanne).

[110] Wang N, Gao J, Jia M, Ma X, Lei Z, Da F, Yan F, Zhang H, Zhou Y, Li M, He G, Meng J, Luo X (2017). Exendin-4 induces bone marrow stromal cells migration through bone marrow-derived macrophages polarization via PKA-STAT3 signaling pathway. Cell Physiol Biochem.

[111] Liu A, Jin S, Fu C, Cui S, Zhang T, Zhu L, Wang Y, Shen SGF, Jiang N, Liu Y (2020). Macrophage-derived small extracellular vesicles promote biomimetic mineralized collagen-mediated endogenous bone regeneration. Int J Oral Sci.

[112] Kechagia JZ, Ivaska J, Roca-Cusachs P (2019). Integrins as biomechanical sensors of the microenvironment. Nat Rev Mol Cell Biol.

[113] Nicolaidou V, Wong MM, Redpath AN, Ersek A, Baban DF, Williams LM, Cope AP, Horwood NJ (2012). Monocytes induce STAT3 activation in human mesenchymal stem cells to promote osteoblast formation. PLoS ONE.

[114] Xiao L, Zhou Y, Zhu L, Yang S, Huang R, Shi W, Peng B, Xiao Y (2018). SPHK1-S1PR1-RANKL Axis regulates the interactions between macrophages and BMSCs in inflammatory bone loss. J Bone Miner Res.

[115] Tu B, Liu S, Liu G, Yan W, Wang Y, Li Z, Fan C (2015). Macrophages derived from THP-1 promote the osteogenic differentiation of mesenchymal stem cells through the IL-23/IL-23R/β-catenin pathway. Exp Cell Res.

[116] Wang J, Yan S, Lu H, Wang S, Xu D (2019). METTL3 attenuates LPS-induced inflammatory response in macrophages via NF-*κ*B signaling pathway. Mediators Inflamm.

[117] Liu Y, Liu Z, Tang H, Shen Y, Gong Z, Xie N, Zhang X, Wang W, Kong W, Zhou Y, Fu Y (2019). The *N*6-methyladenosine (m6A)-forming enzyme METTL3 facilitates M1 macrophage polarization through the methylation of *STAT1* mRNA. Am J Physiol Cell Physiol.

[118] Fu X, Han B, Cai S, Lei Y, Sun T, Sheng Z (2009). Migration of bone marrow-derived mesenchymal stem cells induced by tumor necrosis factor-alpha and its possible role in wound healing. Wound Repair Regen.

[119] Anton K, Banerjee D, Glod J (2012). Macrophage-associated mesenchymal stem cells assume an activated, migratory, pro-inflammatory phenotype with increased IL-6 and CXCL10 secretion. PLoS ONE.

[120] Yan G, Yuan Y, He M, Gong R, Lei H, Zhou H, Wang W, Du W, Ma T, Liu S, Xu Z, Gao M, Yu M, Bian Y, Pang P, Li X, Yu S, Yang F, Cai B, Yang L (2020). m6A methylation of precursor-miR-320/RUNX2 controls osteogenic potential of bone marrow-derived mesenchymal stem cells. Mol Ther Nucleic Acids.

[121] Qiao W, Wong KHM, Shen J, Wang W, Wu J, Li J, Lin Z, Chen Z, Matinlinna JP, Zheng Y, Wu S, Liu X, Lai KP, Chen Z, Lam YW, Cheung KMC, Yeung KWK (2021). TRPM7 kinase-mediated immunomodulation in macrophage plays a central role in magnesium ion-induced bone regeneration. Nat Commun.

[122] Córdova LA, Loi F, Lin TH, Gibon E, Pajarinen J, Nabeshima A, Lu L, Yao Z, Goodman SB (2017). CCL2, CCL5, and IGF-1 participate in the immunomodulation of osteogenesis during M1/M2 transition in vitro. J Biomed Mater Res A.

[123] Yoon DS, Lee KM, Kim SH, Kim SH, Jung Y, Kim SH, Park KH, Choi Y, Ryu HA, Choi WJ, Lee JW (2016). Synergistic action of IL-8 and bone marrow concentrate on cartilage regeneration through upregulation of chondrogenic transcription factors. Tissue Eng Part A.

[124] Medina RJ, O'Neill CL, O'Doherty TM, Knott H, Guduric-Fuchs J, Gardiner TA, Stitt AW (2011). Myeloid angiogenic cells act as alternative M2 macrophages and modulate angiogenesis through interleukin-8. Mol Med.

[125] Yang A, Lu Y, Xing J, Li Z, Yin X, Dou C, Dong S, Luo F, Xie Z, Hou T, Xu J (2018). IL-8 enhances therapeutic effects of BMSCs on bone regeneration via CXCR2-mediated PI3k/Akt signaling pathway. Cell Physiol Biochem.

[126] Marques RE, Guabiraba R, Russo RC, Teixeira MM (2013). Targeting CCL5 in inflammation. Expert Opin Ther Targets.

[127] Xiong Y, Chen L, Yan C, Zhou W, Yu T, Sun Y, Cao F, Xue H, Hu Y, Chen D, Mi B, Liu G (2020). M2 Macrophagy-derived exosomal miRNA-5106 induces bone mesenchymal stem cells towards osteoblastic fate by targeting salt-inducible kinase 2 and 3. J Nanobiotechnology.

[128] Zhang B, Li Y, Yu Y, Zhao J, Ou Y, Chao Y, Yang B, Yu X (2018). MicroRNA-378 promotes osteogenesis-angiogenesis coupling in BMMSCs for potential bone regeneration. Anal Cell Pathol (Amst).

[129] Qi Y, Zhu T, Zhang T, Wang X, Li W, Chen D, Meng H, An S (2021). M1 macrophage-derived exosomes transfer miR-222 to induce bone marrow mesenchymal stem cell apoptosis. Lab Invest.

[130] Tepper OM, Capla JM, Galiano RD, Ceradini DJ, Callaghan MJ, Kleinman ME, Gurtner GC (2005). Adult vasculogenesis occurs through in situ recruitment, proliferation, and tubulization of circulating bone marrow-derived cells. Blood.

[131] Zaidi N, Nixon AJ (2007). Stem cell therapy in bone repair and regeneration. Ann N Y Acad Sci.

[132] Klimczak A, Kozlowska U, Kurpisz M (2018). Muscle stem/progenitor cells and mesenchymal stem cells of bone marrow origin for skeletal muscle regeneration in muscular dystrophies. Arch Immunol Ther Exp (Warsz).

[133] Cashman TJ, Gouon-Evans V, Costa KD (2013). Mesenchymal stem cells for cardiac therapy: practical challenges and potential mechanisms. Stem Cell Rev Rep.

[134] Wang Y, Han ZB, Song YP, Han ZC (2012). Safety of mesenchymal stem cells for clinical application. Stem Cells Int.

[135] Woodbury D, Schwarz EJ, Prockop DJ, Black IB (2000). Adult rat and human bone marrow stromal cells differentiate into neurons. J Neurosci Res.

[136] Wakitani S, Saito T, Caplan AI (1995). Myogenic cells derived from rat bone marrow mesenchymal stem cells exposed to 5-azacytidine. Muscle Nerve.

[137] Oswald J, Boxberger S, Jørgensen B, Feldmann S, Ehninger G, Bornhäuser M, Werner C (2004). Mesenchymal stem cells can be differentiated into endothelial cells in vitro. Stem Cells.

[138] Squillaro T, Peluso G, Galderisi U (2016). Clinical trials with mesenchymal stem cells: an update. Cell Transplant.

[139] Almalki SG, Agrawal DK (2016). Key transcription factors in the differentiation of mesenchymal stem cells. Differentiation.

[140] Lin H, Sohn J, Shen H, Langhans MT, Tuan RS (2019). Bone marrow mesenchymal stem cells: Aging and tissue engineering applications to enhance bone healing. Biomaterials.

[141] Aykan A, Ozturk S, Sahin I, Gurses S, Ural AU, Oren NC, Isik S (2013). Biomechanical analysis of the effect of mesenchymal stem cells on mandibular distraction osteogenesis. J Craniofac Surg.

[142] Field JR, McGee M, Stanley R, Ruthenbeck G, Papadimitrakis T, Zannettino A, Gronthos S, Itescu S (2011). The efficacy of allogeneic mesenchymal precursor cells for the repair of an ovine tibial segmental defect. Vet Comp Orthop Traumatol.

[143] Niemeyer P, Schönberger TS, Hahn J, Kasten P, Fellenberg J, Suedkamp N, Mehlhorn AT, Milz S, Pearce S (2010). Xenogenic transplantation of human mesenchymal stem cells in a critical size defect of the sheep tibia for bone regeneration. Tissue Eng Part A.

[144] Ruan SQ, Deng J, Yan L, Huang WL (2018). Composite scaffolds loaded with bone mesenchymal stem cells promote the repair of radial bone defects in rabbit model. Biomed Pharmacother.

[145] Liu Q, Wen Y, Qiu J, Zhang Z, Jin Z, Cao M, Jiao Y, Yang H (2020). Local SDF-1α application enhances the therapeutic efficacy of BMSCs transplantation in osteoporotic bone healing. Heliyon.

[146] Hernigou P, Poignard A, Manicom O, Mathieu G, Rouard H (2005). The use of percutaneous autologous bone marrow transplantation in nonunion and avascular necrosis of bone. J Bone Joint Surg Br.

[147] Kolf CM, Cho E, Tuan RS (2007). Mesenchymal stromal cells: Biology of adult mesenchymal stem cells: regulation of niche, self-renewal and differentiation. Arthritis Res Ther.

[148] De Becker A, Riet IV (2016). Homing and migration of mesenchymal stromal cells: How to improve the efficacy of cell therapy?. World J Stem Cells.

[149] Toupadakis CA, Wong A, Genetos DC, Chung DJ, Murugesh D, Anderson MJ, Loots GG, Christiansen BA, Kapatkin AS, Yellowley CE (2012). Long-term administration of AMD3100, an antagonist of SDF-1/CXCR4 signaling, alters fracture repair. J Orthop Res.

[150] Kolar P, Gaber T, Perka C, Duda GN, Buttgereit F (2011). Human early fracture hematoma is characterized by inflammation and hypoxia. Clin Orthop Relat Res.

[151] Komori T (2011). Signaling networks in RUNX2-dependent bone development. J Cell Biochem.

[152] Wang C, Li Y, Yang M, Zou Y, Liu H, Liang Z, Yin Y, Niu G, Yan Z, Zhang B (2018). Efficient differentiation of bone marrow mesenchymal stem cells into endothelial cells in vitro. Eur J Vasc Endovasc Surg.

[153] Fu L, Zhu L, Huang Y, Lee TD, Forman SJ, Shih CC (2008). Derivation of neural stem cells from mesenchymal stemcells: evidence for a bipotential stem cell population. Stem Cells Dev.

[154] Hermann A, Gastl R, Liebau S, Popa MO, Fiedler J, Boehm BO, Maisel M, Lerche H, Schwarz J, Brenner R, Storch A (2004). Efficient generation of neural stem cell-like cells from adult human bone marrow stromal cells. J Cell Sci.

[155] Kanczler JM, Oreffo RO (2008). Osteogenesis and angiogenesis: the potential for engineering bone. Eur Cell Mater.

[156] Caplan AI (2016). MSCs: the sentinel and safe-guards of injury. J Cell Physiol.

[157] Ogata K, Katagiri W, Hibi H (2017). Secretomes from mesenchymal stem cells participate in the regulation of osteoclastogenesis in vitro. Clin Oral Investig.

[158] Kuroda K, Kabata T, Hayashi K, Maeda T, Kajino Y, Iwai S, Fujita K, Hasegawa K, Inoue D, Sugimoto N, Tsuchiya H (2015). The paracrine effect of adipose-derived stem cells inhibits osteoarthritis progression. BMC Musculoskelet Disord.

[159] Chen S, Liang H, Ji Y, Kou H, Zhang C, Shang G, Shang C, Song Z, Yang L, Liu L, Wang Y, Liu H (2021). Curcumin modulates the crosstalk between macrophages and bone mesenchymal stem cells to ameliorate osteogenesis. Front Cell Dev Biol.

[160] Clarke B (2008). Normal bone anatomy and physiology. Clin J Am Soc Nephrol.

[161] Gao X, Usas A, Proto JD, Lu A, Cummins JH, Proctor A, Chen CW, Huard J (2014). Role of donor and host cells in muscle-derived stem cell-mediated bone repair: differentiation vs. paracrine effects. FASEB J.

[162] Hung SC, Pochampally RR, Chen SC, Hsu SC, Prockop DJ (2007). Angiogenic effects of human multipotent stromal cell conditioned medium activate the PI3K-Akt pathway in hypoxic endothelial cells to inhibit apoptosis, increase survival, and stimulate angiogenesis. Stem Cells.

[163] Shin Y, Won Y, Yang JI, Chun JS (2019). CYTL1 regulates bone homeostasis in mice by modulating osteogenesis of mesenchymal stem cells and osteoclastogenesis of bone marrow-derived macrophages. Cell Death Dis.

[164] Waterman RS, Tomchuck SL, Henkle SL, Betancourt AM (2010). A new mesenchymal stem cell (MSC) paradigm: polarization into a pro-inflammatory MSC1 or an Immunosuppressive MSC2 phenotype. PLoS ONE.

[165] Gu YZ, Xue Q, Chen YJ, Yu GH, Qing MD, Shen Y, Wang MY, Shi Q, Zhang XG (2013). Different roles of PD-L1 and FasL in immunomodulation mediated by human placenta-derived mesenchymal stem cells. Hum Immunol.

[166] Augello A, Tasso R, Negrini SM, Amateis A, Indiveri F, Cancedda R, Pennesi G (2005). Bone marrow mesenchymal progenitor cells inhibit lymphocyte proliferation by activation of the programmed death 1 pathway. Eur J Immunol.

[167] de Castro LL, Lopes-Pacheco M, Weiss DJ, Cruz FF, Rocco PRM (2019). Current understanding of the immunosuppressive properties of mesenchymal stromal cells. J Mol Med (Berl).

[168] Khodayari S, Khodayari H, Amiri AZ, Eslami M, Farhud D, Hescheler J, Nayernia K (2019). Inflammatory microenvironment of acute myocardial infarction prevents regeneration of heart with stem cells therapy. Cell Physiol Biochem.

[169] Cho DI, Kim MR, Jeong HY, Jeong HC, Jeong MH, Yoon SH, Kim YS, Ahn Y (2014). Mesenchymal stem cells reciprocally regulate the M1/M2 balance in mouse bone marrow-derived macrophages. Exp Mol Med.

[170] Zhao J, Li X, Hu J, Chen F, Qiao S, Sun X, Gao L, Xie J, Xu B (2019). Mesenchymal stromal cell-derived exosomes attenuate myocardial ischaemia-reperfusion injury through miR-182-regulated macrophage polarization. Cardiovasc Res.

[171] Maggini J, Mirkin G, Bognanni I, Holmberg J, Piazzón IM, Nepomnaschy I, Costa H, Cañones C, Raiden S, Vermeulen M, Geffner JR (2010). Mouse bone marrow-derived mesenchymal stromal cells turn activated macrophages into a regulatory-like profile. PLoS ONE.

[172] Weiss ARR, Dahlke MH (2019). Immunomodulation by mesenchymal stem cells (MSCs): mechanisms of action of living, apoptotic, and dead MSCs. Front Immunol.

[173] Zhao H, Shang Q, Pan Z, Bai Y, Li Z, Zhang H, Zhang Q, Guo C, Zhang L, Wang Q (2018). Exosomes from adipose-derived stem cells attenuate adipose inflammation and obesity through polarizing M2 macrophages and Beiging in white adipose tissue. Diabetes.

[174] Zhang J, Rong Y, Luo C, Cui W (2020). Bone marrow mesenchymal stem cell-derived exosomes prevent osteoarthritis by regulating synovial macrophage polarization. Aging (Albany NY).

[175] He X, Dong Z, Cao Y, Wang H, Liu S, Liao L, Jin Y, Yuan L, Li B (2019). MSC-derived exosome promotes M2 polarization and enhances cutaneous wound healing. Stem Cells Int.

[176] Mao G, Zhang Z, Hu S, Zhang Z, Chang Z, Huang Z, Liao W, Kang Y (2018). Exosomes derived from miR-92a-3p-overexpressing human mesenchymal stem cells enhance chondrogenesis and suppress cartilage degradation via targeting WNT5A. Stem Cell Res Ther.

[177] Yin Y, Li X, He XT, Wu RX, Sun HH, Chen FM (2017). Leveraging stem cell homing for therapeutic regeneration. J Dent Res.

[178] Brown BN, Sicari BM, Badylak SF (2014). Rethinking regenerative medicine: a macrophage-centered approach. Front Immunol.

[179] Sadowska JM, Wei F, Guo J, Guillem-Marti J, Lin Z, Ginebra MP, Xiao Y (2019). The effect of biomimetic calcium deficient hydroxyapatite and sintered β-tricalcium phosphate on osteoimmune reaction and osteogenesis. Acta Biomater.

[180] Trindade R, Albrektsson T, Tengvall P, Wennerberg A (2016). Foreign body reaction to biomaterials: on mechanisms for buildup and breakdown of osseointegration. Clin Implant Dent Relat Res.

[181] Le Blanc K, Mougiakakos D (2012). Multipotent mesenchymal stromal cells and the innate immune system. Nat Rev Immunol.

[182] Vishwakarma A, Bhise NS, Evangelista MB, Rouwkema J, Dokmeci MR, Ghaemmaghami AM, Vrana NE, Khademhosseini A (2016). Engineering immunomodulatory biomaterials to tune the inflammatory response. Trends Biotechnol.

[183] Chen Z, Wu C, Gu W, Klein T, Crawford R, Xiao Y (2014). Osteogenic differentiation of bone marrow MSCs by β-tricalcium phosphate stimulating macrophages via BMP2 signalling pathway. Biomaterials.

[184] Rh Owen G, Dard M, Larjava H (2018). Hydoxyapatite/beta-tricalcium phosphate biphasic ceramics as regenerative material for the repair of complex bone defects. J Biomed Mater Res B Appl Biomater.

[185] Zheng M, Weng M, Zhang X, Li R, Tong Q, Chen Z (2021). Beta-tricalcium phosphate promotes osteogenic differentiation of bone marrow-derived mesenchymal stem cells through macrophages. Biomed Mater.

[186] Chen Z, Yuen J, Crawford R, Chang J, Wu C, Xiao Y (2015). The effect of osteoimmunomodulation on the osteogenic effects of cobalt incorporated β-tricalcium phosphate. Biomaterials.

[187] Ujiie Y, Karakida T, Yamakoshi Y, Ohshima T, Gomi K, Oida S (2016). Interleukin-4 released from human gingival fibroblasts reduces osteoclastogenesis. Arch Oral Biol.

[188] Gamblin AL, Brennan MA, Renaud A, Yagita H, Lézot F, Heymann D, Trichet V, Layrolle P (2014). Bone tissue formation with human mesenchymal stem cells and biphasic calcium phosphate ceramics: the local implication of osteoclasts and macrophages. Biomaterials.

[189] Tasso R, Ulivi V, Reverberi D, Lo Sicco C, Descalzi F, Cancedda R (2013). In vivo implanted bone marrow-derived mesenchymal stem cells trigger a cascade of cellular events leading to the formation of an ectopic bone regenerative niche. Stem Cells Dev.

[190] Tour G, Wendel M, Tcacencu I (2014). Bone marrow stromal cells enhance the osteogenic properties of hydroxyapatite scaffolds by modulating the foreign body reaction. J Tissue Eng Regen Med.

[191] Seebach E, Freischmidt H, Holschbach J, Fellenberg J, Richter W (2014). Mesenchymal stroma cells trigger early attraction of M1 macrophages and endothelial cells into fibrin hydrogels, stimulating long bone healing without long-term engraftment. Acta Biomater.

[192] Li K, Xue Y, Zhou J, Han J, Zhang L, Han Y (2020). Silanized NaCa2HSi3O9 nanorods with a reduced pH increase on Ti for improving osteogenesis and angiogenesis in vitro. J Mater Chem B.

[193] Zhu S, Chen P, Chen Y, Li M, Chen C, Lu H (2020). 3D-printed extracellular matrix/polyethylene glycol diacrylate hydrogel incorporating the anti-inflammatory phytomolecule honokiol for regeneration of osteochondral defects. Am J Sports Med.

[194] Engler AJ, Sen S, Sweeney HL, Discher DE (2006). Matrix elasticity directs stem cell lineage specification. Cell.

[195] Cipitria A, Boettcher K, Schoenhals S, Garske DS, Schmidt-Bleek K, Ellinghaus A, Dienelt A, Peters A, Mehta M, Madl CM, Huebsch N, Mooney DJ, Duda GN (2017). In-situ tissue regeneration through SDF-1α driven cell recruitment and stiffness-mediated bone regeneration in a critical-sized segmental femoral defect. Acta Biomater.

[196] Sussman EM, Halpin MC, Muster J, Moon RT, Ratner BD (2014). Porous implants modulate healing and induce shifts in local macrophage polarization in the foreign body reaction. Ann Biomed Eng.

[197] Akan Z, Aksu B, Tulunay A, Bilsel S, Inhan-Garip A (2010). Extremely low-frequency electromagnetic fields affect the immune response of monocyte-derived macrophages to pathogens. Bioelectromagnetics.

[198] Hoare JI, Rajnicek AM, McCaig CD, Barker RN, Wilson HM (2016). Electric fields are novel determinants of human macrophage functions. J Leukoc Biol.

[199] Dai X, Heng BC, Bai Y, You F, Sun X, Li Y, Tang Z, Xu M, Zhang X, Deng X (2020). Restoration of electrical microenvironment enhances bone regeneration under diabetic conditions by modulating macrophage polarization. Bioact Mater.

[200] Mahbub S, Deburghgraeve CR, Kovacs EJ (2012). Advanced age impairs macrophage polarization. J Interferon Cytokine Res.

[201] Barrett JP, Costello DA, O'Sullivan J, Cowley TR, Lynch MA (2015). Bone marrow-derived macrophages from aged rats are more responsive to inflammatory stimuli. J Neuroinflammation.

[202] Hubbard RE, Woodhouse KW (2010). Frailty, inflammation and the elderly. Biogerontology.

[203] Baht GS, Silkstone D, Vi L, Nadesan P, Amani Y, Whetstone H, Wei Q, Alman BA (2015). Exposure to a youthful circulaton rejuvenates bone repair through modulation of β-catenin. Nat Commun.

[204] Ambrosi TH, Scialdone A, Graja A, Gohlke S, Jank AM, Bocian C, Woelk L, Fan H, Logan DW, Schürmann A, Saraiva LR, Schulz TJ (2017). Adipocyte accumulation in the bone marrow during obesity and aging impairs stem cell-based hematopoietic and bone regeneration. Cell Stem Cell.

[205] Marędziak M, Marycz K, Tomaszewski KA, Kornicka K, Henry BM (2016). The influence of aging on the regenerative potential of human adipose derived mesenchymal stem cells. Stem Cells Int.

[206] Kim J, Ko J (2014). A novel PPARγ2 modulator sLZIP controls the balance between adipogenesis and osteogenesis during mesenchymal stem cell differentiation. Cell Death Differ.

