# Correction: The crosstalk between macrophages and bone marrow mesenchymal stem cells in bone healing

**DOI:** 10.1186/s13287-022-03211-5

**Published:** 2022-12-17

**Authors:** Yu-Hao Wang, Cheng-Zhi Zhao, Ren-Yi Wang, Qian-Xin Du, Ji-Yuan Liu, Jian Pan

**Affiliations:** 1 State Key Laboratory of Oral Disease, West China Hospital of Stomatology, Sichuan University, #14 Third Section, Renmin Road South, Chengdu, 610041 People’s Republic of China; 2 National Clinical Research Center for Oral Diseases and Department of Oral and Maxillofacial Surgery, West China Hospital of Stomatology, Sichuan University, Chengdu, 610041 People’s Republic of China; 3 Chengdu Advanced Medical Science Center, West China Hospital of Stomatology, Sichuan University, Chengdu, 610041 Sichuan Province People’s Republic of China

**Correction: Stem Cell Research & Therapy (2022) 13:511**

10.1186/s13287-022-03199-y

The original article [1] displayed an incorrect Funding number which has since been amended.
